# Sinus node sparing novel hybrid approach for treatment of inappropriate sinus tachycardia/postural sinus tachycardia: multicenter experience

**DOI:** 10.1007/s10840-021-01044-5

**Published:** 2021-08-23

**Authors:** Carlo de Asmundis, Gian-Battista Chierchia, Dhanunjaya Lakkireddy, Ahmed Romeya, Eric Okum, Gaurang Gandhi, Juan Sieira, Margot Vloka, Stephen D. Jones, Hemal Shah, Marshall Winner, Dilesh Patel, S. Patrick Whalen, Elijah H. Beaty, Edward Hal Kincaid, Anson Lee, Chad Brodt, Benadict J. Taylor, Ilyas Colombowala, Matthew Romano, Fred Morady, Erwin Ströker, Ingrid Overeinder, Gezim Bala, Justin Van Meeteren, Yoaav Krauthammer, Scott Koerber, Christian Shults, Athanasios Thomaides, Nitish Badhwar, Rakesh Gopinathannair, Alap Shah, Rangarao Tummala, David Bello, Steve Hoff, Alexandre Almorad, Kenneth Frazier, Pedro Brugada, Mark La Meir

**Affiliations:** 1 Heart Rhythm Management Center, Postgraduate Program in Cardiac Electrophysiology and Pacing, Universitair Ziekenhuis Brussel-Vrije Universiteit Brussel, Laarbeeklaan 101, 1090 Brussels, Belgium; 2 Overland Park Regional Medical Center and Research, Kansas City, KS USA; 3 TriHealth Heart Institute/Bethesda North Hospital, Cincinnati, OH USA; 4 Saint Alphonsus Regional Medical Center, Boise, ID USA; 5 Wake Forest Baptist Health, Winston-Salem, NC USA; 6 Stanford Health Care, University of Stanford, Stanford, CA USA; 7 University of Michigan, Ann Arbor, MI USA; 8 Research Medical Center, Kansas City, MO USA; 9 MedStar Washington Hospital Center, Washington, DC USA; 10 Orlando Regional Medical Center, Orlando, FL US; 11 Cardiac Surgery Department, Universitair Ziekenhuis Brussel-Vrije Universiteit Brussel, Brussels, Belgium

**Keywords:** Inappropriate sinus tachycardia, Postural orthostatic tachycardia, Arrhythmias ablation, Hybrid therapy, Hybrid ablation, Sinus node

## Abstract

**Background:**

The ideal treatment of inappropriate sinus tachycardia (IST) and postural orthostatic tachycardia syndrome (POTS) still needs to be defined. Medical treatments yield suboptimal results. Endocardial catheter ablation of the sinus node (SN) may risk phrenic nerve damage and open-heart surgery may be accompanied by unjustified invasive risks.

**Methods:**

We describe our first multicenter experience of 255 consecutive patients (235 females, 25.94 ± 3.84 years) having undergone a novel SN sparing hybrid thoracoscopic ablation for drug-resistant IST (*n* = 204, 80%) or POTS (*n* = 51, 20%). As previously described, the SN was identified with 3D mapping. Surgery was performed through three 5-mm ports from the right side. A minimally invasive approach with a bipolar radiofrequency clamp was used to ablate targeted areas while sparing the SN region. The targeted areas included isolation of the superior and the inferior caval veins, and a crista terminalis line was made. All lines were interconnected.

**Results:**

Normal sinus rhythm (SR) was restored in all patients at the end of the procedure. All patients discontinued medication during the follow-up. After a blanking period of 6 months, all patients presented stable SR. At a mean of 4.07 ± 1.8 years, normal SN reduction and chronotropic response to exercise were present. In the 51 patients initially diagnosed with POTS, no syncope occurred. During follow-up, pericarditis was the most common complication (121 patients: 47%), with complete resolution in all cases. Pneumothorax was observed in 5 patients (1.9%), only 3 (1.1%) required surgical drainage. Five patients (1.9%) required a dual-chamber pacemaker due to sinus arrest > 5 s.

**Conclusions:**

Preliminary results of this multicenter experience with a novel SN sparing hybrid ablation of IST/POTS, using surgical thoracoscopic video-assisted epicardial ablation combined with simultaneous endocardial 3D mapping may prove to be an efficient and safe therapeutic option in patients with symptomatic drug-resistant IST and POTS. Importantly, in our study, all patients had a complete resolution of the symptoms and restored normal SN activity.

## Introduction

Postural orthostatic tachycardia syndrome (POTS) is a systemic illness, with postural tachycardia being one of several criteria. It is usually characterized by frequent symptoms that occur while standing, such as light-headedness, palpitations, tremor, generalized weakness, blurred vision, exercise intolerance, and fatigue. These symptoms are associated with an increase in heart rate (HR) of > 30 beats per min (bpm) when moving from a recumbent to a standing position (or > 40 bpm in individuals 12 to 19 years of age) and the absence of orthostatic hypotension (> 20-mmHg drop in systolic blood pressure). The standing (or orthostatic) HR of individuals with POTS is often > 120 bpm and increases more in the morning than in the evening [1]. Inappropriate sinus tachycardia (IST) is defined as a sinus HR > 100 bpm at rest (with a mean 24-h HR > 90 bpm not due to primary causes) and is associated with distressing symptoms of palpitations. The prevalence of IST is estimated to be 1.16%, based on a study by Still et al., which evaluated both symptomatic and asymptomatic middle-aged patients [2]. IST is believed to be a chronic condition, but whether and how quickly patients improve is unknown. Patients with IST and POTS require significant care and attention due to the nearly ubiquitous psychosocial distress and the complexity of their problems.

Although the inappropriateness in sinus rate and frequent symptoms of palpitations bring the evaluation focus primarily on cardiovascular indices, frequent multisystem complaints from these patients suggest that IST encompasses a heterogeneous population with extra cardiovascular pathophysiologic mechanisms. Separately described, although with significant overlap in clinical presentation as in patients with IST, is a group of patients known as POTS, in whom the symptoms and tachycardia predominantly develop in the upright position. Clinical presentation and underlying pathophysiology of POTS can be attributed, at least in part, to autonomic dysregulation. Although the precise relationship of IST and POTS is not known, overlapping multisystem, symptoms, and autonomic abnormalities observed in selected patients with IST suggest these two syndrome complexes may share some common pathophysiologic mechanisms. [3]

Medical treatment of IST or POTS has shown suboptimal results. If *β*-blockers and calcium channel blockers are ineffective, ivabradine, an inhibitor of the hyperpolarizing sodium current, may be an option [4].

Radiofrequency sinus node (SN) modification for POTS is not recommended. It often worsens symptoms and occasionally results in permanent phrenic nerve (PN) paralysis or transient superior vena cava syndrome and/or the patient requiring a permanent pacemaker. SN modification, surgical ablation, and sympathetic denervation are not recommended as a part of routine care for patients with IST. Several groups have described modification or ablation of the SN for IST. In general, acute success rates are reasonable, but there is a high rate of symptom recurrence and significant complication rates. In addition, SN modification or ablation does not relieve all IST-associated symptoms. There is also no agreement on the optimal interventional approach; modification or ablation utilizing open chest ablation vs. conventional intravascular access with mapping and ablation. Finally, there is no long-term evidence of symptomatic improvement. Patients and referring physicians need to be aware that despite the potentially invalidating symptoms and the patients’ high motivation, the consequences of open-heart surgery therapy might seriously outweigh any potential benefit [1].

We describe a possible novel substrate for POTS and IST and a novel hybrid ablation technique targeting this substrate. The hypothesis about our ablation strategy is based on consideration regarding embryological heart development. At the early stage of embryogenesis, when the heart is still a tube structure, all cardiomyocytes can automatically initiate impulses. Afterward, the rest of the sinus horn (embryological precursors of the SN, SVC, crista terminalis, and IVC) finally develops into the sinus venosus of the right atrium, a part of the superior and inferior vena cava and the coronary sinus.

## Methods

### Patient population

From June 2015 to June 2020, 255 consecutive patients underwent a novel hybrid ablation for IST or POTS in cardiac centers; the centers were the following: (1) Cardiovascular Center, Universitair Ziekenhuis Brussel-Vrije Universiteit Brussel, Brussels, Belgium; (2) Overland Park Regional Medical Center and Research, Kansas City, KS, USA; (3) TriHealth Heart Institute/Bethesda North Hospital, Cincinnati, OH, USA; (4)Saint Alphonsus Regional Medical Center, Boise, ID, USA; (5) Wake Forest Baptist Health, Winston-Salem, NC, USA; (6) Stanford Health Care, University of Stanford, California; (7) University of Michigan, Ann Arbor, MI, USA; (8) Research Medical Center, Kansas City, MO, USA; (9) MedStar Washington Hospital Center, Washington, DC, USA; and (10) Orlando Regional Medical Center, Orlando, FL, USA. Data were collected in a retrospective fashion.

Diagnosis of IST or POTS was made by following the HRS expert consensus statement on POTS/IST [1]. The patients were evaluated by a cardiologist or together with a neurologist with both being experts in IST and POTS [4]. Primary causes of sinus tachycardia were ruled out, as were other mechanisms of supraventricular tachycardia. Every patient underwent a 12-lead ECG to confirm normal P-wave morphology and a Holter 24-h ECG to evaluate the heart rate variability (HRV) and the circadian variation. Additional evaluations included baseline blood testing (complete metabolic panel, thyroid function, blood count, renal function, electrolytes, drug testing, and serum and urine catecholamines) and autonomic testing and/or long-term monitoring (Loop Holter, implantable Reveal). Autonomic tests were performed in a majority of the patients (212 patients, 83%) according to standard protocols using the Nexfin HD monitor (BMEYE, Amsterdam, The Netherlands) for continuous noninvasive blood pressure measurement. Six cardiovascular reflex tests were collected in the following order with 15 min of recovery time between recordings: (1) expiration-to-inspiration ratio of RR interval during slow deep breathing (heart rate variation); (2) maximum-to-minimum 30-to-15 ratio of RR intervals during active standing; (3) systolic blood pressure response to standing; (4) cold face test; (5) maximum-to-minimum ratio of RR intervals during Valsalva maneuvers (Valsalva ratio); and (6) blood pressure response to sustained handgrip (handgrip ratio). Methodologies, as well as databases of normal values for healthy individuals of these tests, have been described in detail elsewhere [5, 8]. Patients who proved refractory to or intolerant of pharmacologic therapy were offered hybrid ablative treatment. Risks, benefits, and alternatives were thoroughly discussed. All individuals provided written informed consent before the procedure. Data were reviewed under an institutional review board–approved registry.

### Heart rate variability

The frequency-domain measures of HR variability in the population were analyzed using the methods recommended by the Task Force of the European Society of Cardiology [4]. Spectral power was quantified by fast Fourier transform analysis in two frequency bands 0.01 to 0.15 Hz (low frequency [LF]) and 0.15 to 0.40 Hz (high frequency [HF]) [6, 7]. The LF and HF components during the 15-min period in supine and sitting positions and standardized walking were calculated as absolute units. LF/HF ratio was used as the index of sympathovagal balance. In all patients with an implantable cardiac loop recording system (Reveal Linq LNQ11, Medtronic, Minneapolis, USA), we obtained long-term monitoring of HRV and HR.

A Holter electrocardiogram was digitalized to 12-bit data at 128 Hz with a scanner (MARS-PC; GE Company, Fairfield, CT), on which QRS complexes were collected automatically. The results were reviewed and any errors in QRS labeling were edited manually. Patients with implantable loop recordings were analyzed automatically by dedicated internal software (Medtronic, Minneapolis, MN, USA). Computations of HRV measurements were performed using ad hoc analytical software (Kubios HRV freeware). Time-domain variables included the mean normal-to-normal RR intervals (mean NN), standard deviations of the normal-to-normal RR intervals (SDNN), standard deviations of 5-min averages of normal RR intervals (SDANN), and root mean square of differences between adjacent normal RR intervals (rMSSD). For frequency-domain analysis, the power spectrum was quantified by fast Fourier transform for the following frequency bands: 0.0033 to < 0.04 Hz (very low frequency, VLF), 0.04 to < 0.15 Hz (low frequency, LF), and 0.15 to 0.4 Hz (high frequency, HF). LF/HF was also calculated. Frequency-domain measurements of HRV were transformed to natural logarithms for their skewed distribution [5].

## Procedure

The procedure starts with endocardial mapping to ascertain the location of the sinus nodal area, then an epicardial hybrid ablation sparing the sinus nodal area. Lastly, we performed endocardial mapping to prove the consistency of the surgical ablation lesions, and only in the presence of gaps we endocardially touch up to ensure a complete lesion set.

### Hybrid ablation procedure

**Fig. 1:**
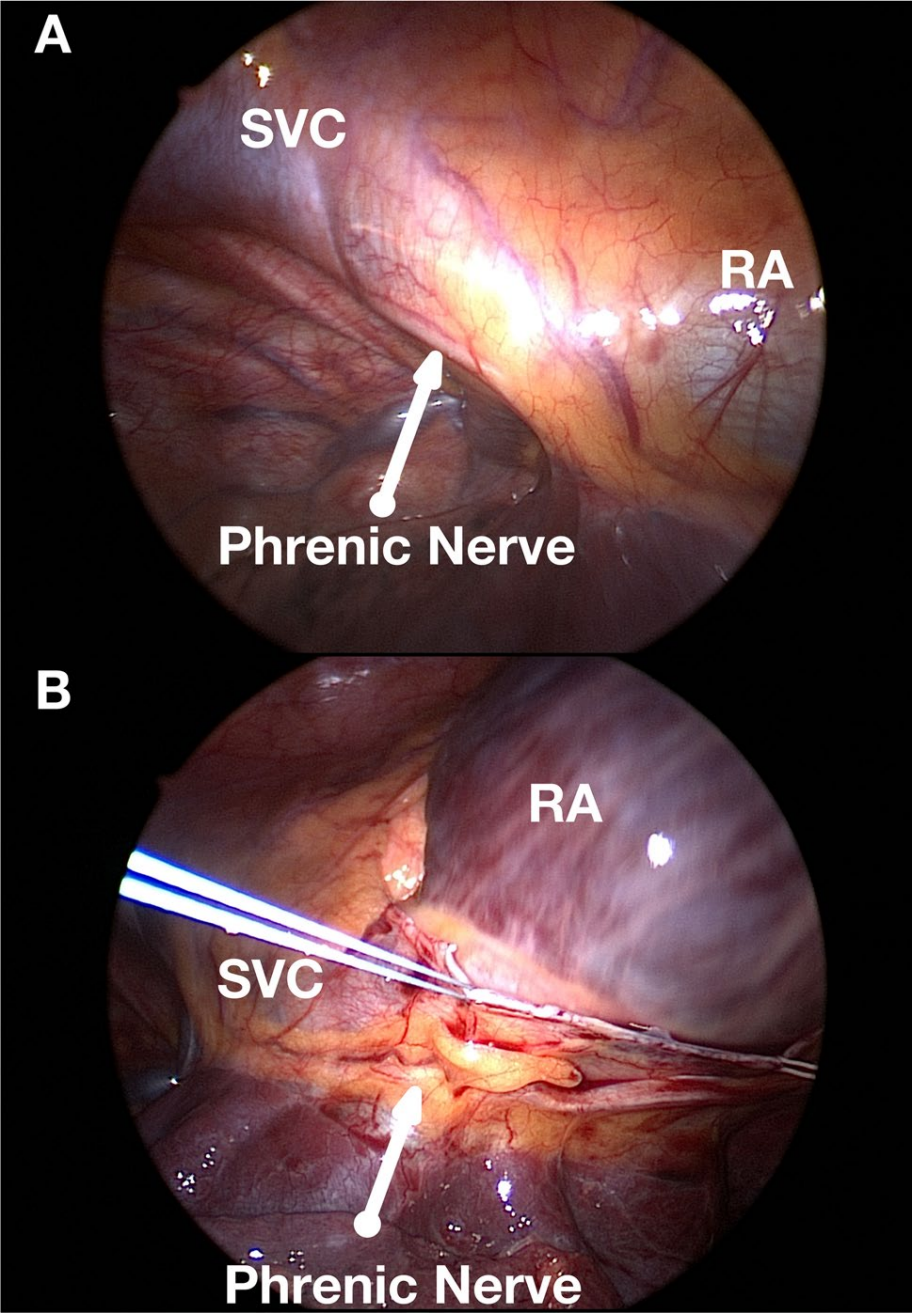
Camera view from the right thoracoscopic approach. SVC, superior vena cava; RA, right atrium**. **Panel **A** is showing the close pericardium view; the schema explains the relation between all different structures; panel** B** is showing the relation modification between the different structures after opening the pericardium

As previously described [8], the right chest was accessed with three 5-mm working ports: a camera port in the fifth intercostal space at the mid-axillary line, a 5-mm port for instruments in the third intercostal space at the anterior axillary line, and a 5-mm port for instruments in the seventh intercostal space at the anterior axillary line. After placement of the camera port, CO_2_ insufflation was initiated at a pressure of 8 mm Hg. This was to increase the working space by pushing the diaphragm down and the heart toward the left. Based on the patient’s presentation and cardiac anatomy, the location of these ports varied. In women, the lateral mammary fold was used. The pericardium was opened with an endoscopic coagulation hook and/or scissors longitudinally, 2 cm anterior to the PN toward the superior caval vein (SVC) and inferior caval vein (IVC) (Fig. 1). To improve visualization and facilitate the dissection of the pericardial reflection of the IVC (to gain access to the oblique sinus), the posterior part of the pericardium was retracted with two sutures that were pulled outside the chest posterior to the camera port. The pericardial reflection of the IVC was bluntly dissected until the opening of the oblique sinus.

After the endocardial mapping of the SN, the SVC and IVC must be free of endovascular catheters and/or the central venous line to avoid damage during clamping and ablation. A bipolar bidirectional RF clamping device (EMR2, AtriCure Inc., Mason, OH, USA) was positioned over the right atrium along the crista terminalis. The posterior jaw of the clamp was positioned in the oblique sinus and the anterior jaw over Waterston’s groove, covering the crista terminalis. Six applications were performed. The bipolar bidirectional RF clamping device was positioned over the IVC at the junction with the right atrium. Care must be taken to avoid damage to adjacent structures, such as the coronary sinus or the right ventricle, by visualization through the oblique sinus. Three ablations were performed while trying to connect this ablation with the lateral crista ablation line. The bipolar clamp was then positioned over the SVC at the junction with the right atrium. The clamp was closed while inspection of the P-wave was closely monitored. Once safe placement was confirmed, two to three applications were performed at this level. This usually reduces the HR by around 30% acutely, but the rate may recover partially after a few minutes. Then, the connection of the ablation lines was confirmed or was re-ablated. Once lines were transmural and connected, this often resulted in a junctional rhythm, which recovered to slow sinus rhythm after minutes. The pericardium was closed, and the right lung was reinflated. We observed a slowing of the HR during the procedure in all patients immediately after the interconnection of the ablation lines. We considered an endpoint of the ablation a reduction of at least 25% of the HR or accelerated junctional rhythm. All cardiac surgeons were performing routinely between 15 and 20 cases of thoracoscopic atrial fibrillation ablation prior to performing these procedures [10].

### Electrophysiological 3D mapping of the sinus node

**Fig. 2:**
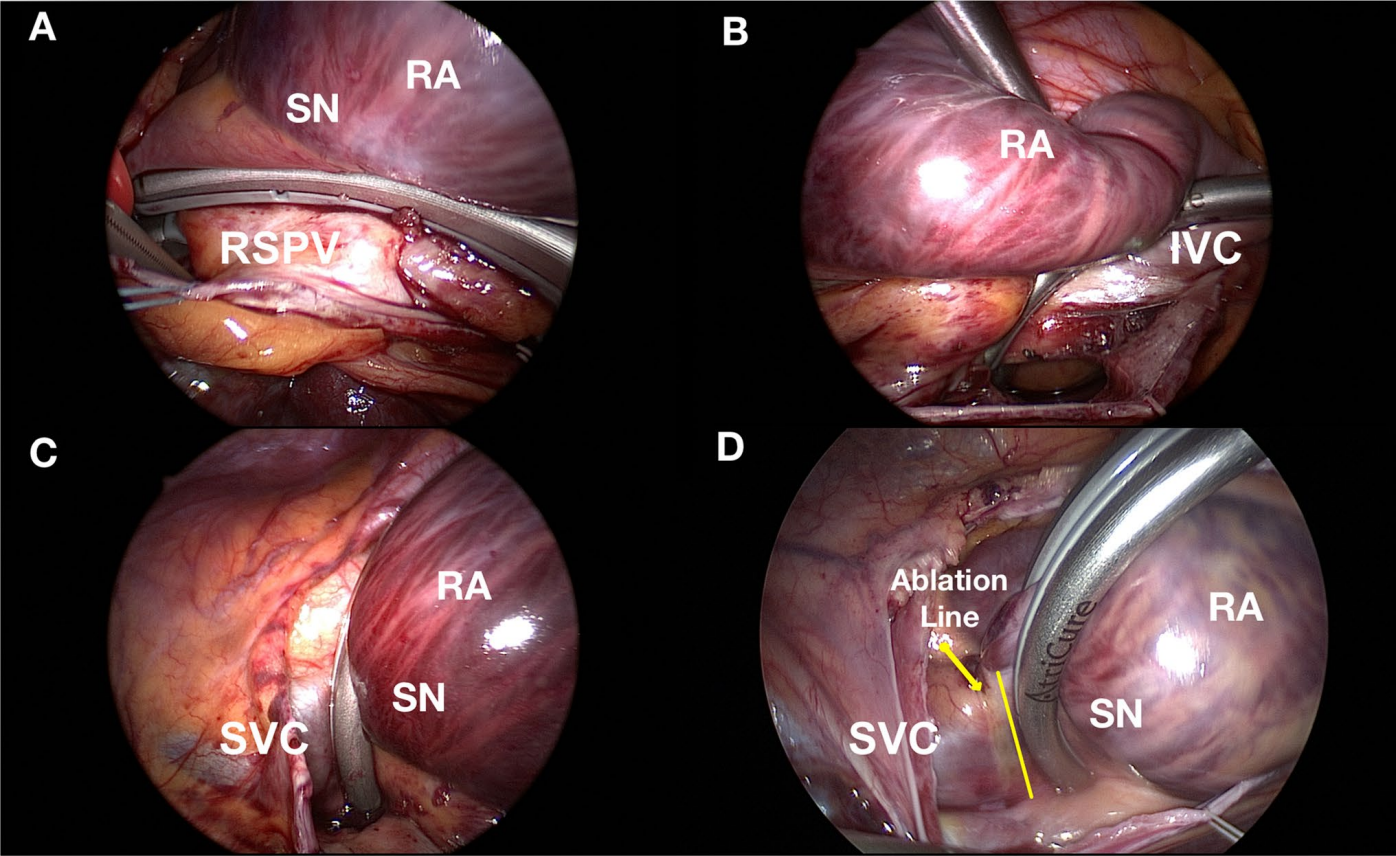
Camera view from the right thoracoscopic approach. SN, sinus node; RA, right atrium; RSPV, right superior pulmonary vein; IVC, inferior vena cava; SVC, superior vena cava. Panel** A** showing the clamping of crista terminalis in relation with SN and RSPV; panel** B** showing the clamping of IVC in relation with RA; panel** C** showing the clamping of SVC in relation with RA and SN; panel** D** showing the second clamping of SVC in relation with RA and SN; the yellow line underlines the previous ablation line

Patients were studied under general anesthesia without the use of paralytic agents. Anti-arrhythmic drugs and beta blockers were withheld for at least five half-lives prior to the procedure. A multipolar catheter was advanced through the right femoral vein into the right atrium (RA) and positioned with its distal 10 poles in the coronary sinus and the proximal poles within the tricuspid annulus. Mapping was performed using a multipolar catheter, either a PentaRay catheter, a circular Optima catheter, or a multipolar basket. A 3-dimensional activation map was created using the appropriate catheter to each mapping system. A baseline electrophysiologic study was performed to exclude other mechanisms of supraventricular tachycardia. Bipolar activation mapping identified the earliest site referenced to both an endocardial fiducial point (e.g., coronary sinus electrogram) and the surface P-wave. During the mapping, the position of the endocardial catheter was observed using the thoracoscopic video system and was marked by the surgeon with methylene blue. Mapping was performed before and after the ablation. Both maps were obtained during single-lung ventilation and open pericardium. The major aim of the mapping was to visualize the sinus node region and the anatomical relation between SVC, crista terminalis, and IVC and guide the surgeons to preserve the sinus node from the lesion set. After ablation, a map of the ablation scar was used to confirm the continuity and transmurality of the ablation lines (Fig. 2).

### Pharmacological prevention treatment of pericarditis

Pericarditis seems to be the most common complication during postoperative recovery as previously described [8]. Therefore, we introduced a preventive pharmacological treatment on day 1 post-intervention to prevent the occurrence of iatrogenic pericarditis in all patients. The empiric treatments were aspirin (750–1000 mg every 8 h, 1–2 weeks; decreasing doses by 250–500 mg every 1–2 weeks) or NSAIDs (ibuprofen 600 mg every 8 h, 1–2 weeks; decreasing doses by 200–400 mg every 1–2 weeks) as mainstays, associated to colchicine at a low, weigh-adjusted dose (0.5 mg once < 70 kg or 0.5 mg twice daily > 70 kg) to improve the response to medical therapy and prevent a recurrence. Corticosteroids should be considered the second option in patients with contraindications and failure of aspirin or NSAIDs because of the risk of favoring the chronic evolution of the disease and promoting drug dependence. In this case, they were used with colchicine. If used, low to moderate doses (i.e., prednisone 0.2–0.5 mg/kg/day or equivalent) were recommended instead of high doses (i.e., prednisone 1.0 mg/kg/day or equivalent). The initial dose was maintained for 2 weeks or until resolution of the symptoms and eventually normalization of C-reactive protein, then tapering was applied [11]. In the population, as previously described [8], a rate of pericarditis of 78% was observed; however, the rate of pericarditis in the population that received a preventative pharmacological treatment regimen improved to 6%.

## Statistical analysis

Continuous variables are expressed as mean ± SD or median and range as appropriate. Categorical variables are expressed as absolute and relative frequencies. Comparisons of continuous variables were done with a Student’s *t*-test or the Mann–Whitney *U* test as appropriate. The chi-square test or the Fisher’s exact test was used to compare categorical variables as appropriate. A *p* value < 0.05 was considered statistically significant. Statistical analyses were conducted using the SPSS software (SPSS v20, Chicago, IL, USA).

## Results

### Patient population and baseline characteristics

Two hundred and fifty-five consecutive patients (age 25.94 ± 3.84 years; female 92%) were included in the study; 204 patients were diagnosed with IST (80%) and 51 patients with POTS (20%). The baseline characteristics of the study population together with the symptoms are detailed in Table 1.

**Table 1:** Baseline characteristics *n* = 255

Female gender (%)	235 (92)
Age (years)	25.94 ± 3.84
Duration of symptoms (months)	40.10 ± 20.22
Inappropriate sinus tachycardia (IST) (%) Postural orthostatic tachycardia (POTS) (%)	204 (80) 51 (20)
Left ventricular ejection fraction (%)	56.71 ± 1.29
Body mass index (kg/m^2^)	22.3 ± 4.1
Symptoms	
Palpitation	247 (97)
Syncope with POTS	51 (20)
Pre-syncope without POTS	25 (10)
Dizziness	198 (77)
Dyspnea	98 (38)
Fatigue	245 (96)
Therapy attempts	
Ivabradine (%) *discontinued due to intolerance*	248 (97) *228 (89)*
Calcium channel blockers (%) *discontinued due to intolerance*	246 (96) 2*46* (96)
IC class anti-arrhythmic drugs (%) *discontinued due to intolerance*	50 (19) *50 (19)*
Beta blockers (%) *discontinued due to intolerance*	70 (27) 70 (27)

Pharmacological treatment was attempted in all patients without benefit. The drugs are detailed in Table 1. One hundred fifty-two (59%) patients underwent a previous electrophysiological study in another center without a diagnosis, 69 patients (27%) underwent a slow pathway ablation for documented AV nodal reentry tachycardia, 15 patients (0.5%) underwent a previously typical right flutter ablation, and only 1 (0.3%) patient presenting episodes of atrial fibrillation was treated by pulmonary vein isolation. Two hundred twenty-four patients (87%) received Reveal LINQ implantations (Table 2).

**Table 2:** Previous electrophysiological procedures *n* = 255

Electrophysiological study without diagnosis (%)	152 (59)
Reveal implantation (%)	224 (87)
AVNRT ablation (%)	69 (27)
Typical right atrial flutter ablation (%)	15 (0.5)
PVI with cryoballoon ablation (%)	1 (0.3)

### Procedural characteristics and inhospital stay

All procedures were performed under general anesthesia. We were able to collect data from 202 procedures where the mean total procedural duration was 173 ± 11 min; mean 3D mapping time was 10 ± 5 min; mean surgical time was 44 ± 18 min. A mean number of 11.5 ± 1.5 applications per patient was performed with the bipolar radiofrequency clamp. The mean duration of hospitalization was 4.04 ± 0.37 days; the mean duration in the intensive care unit was 1.06 ± 0.09 days. All patients had a restored normal sinus function at the end of the procedure.

### Electrophysiological findings during ablation

**Fig. 3:**
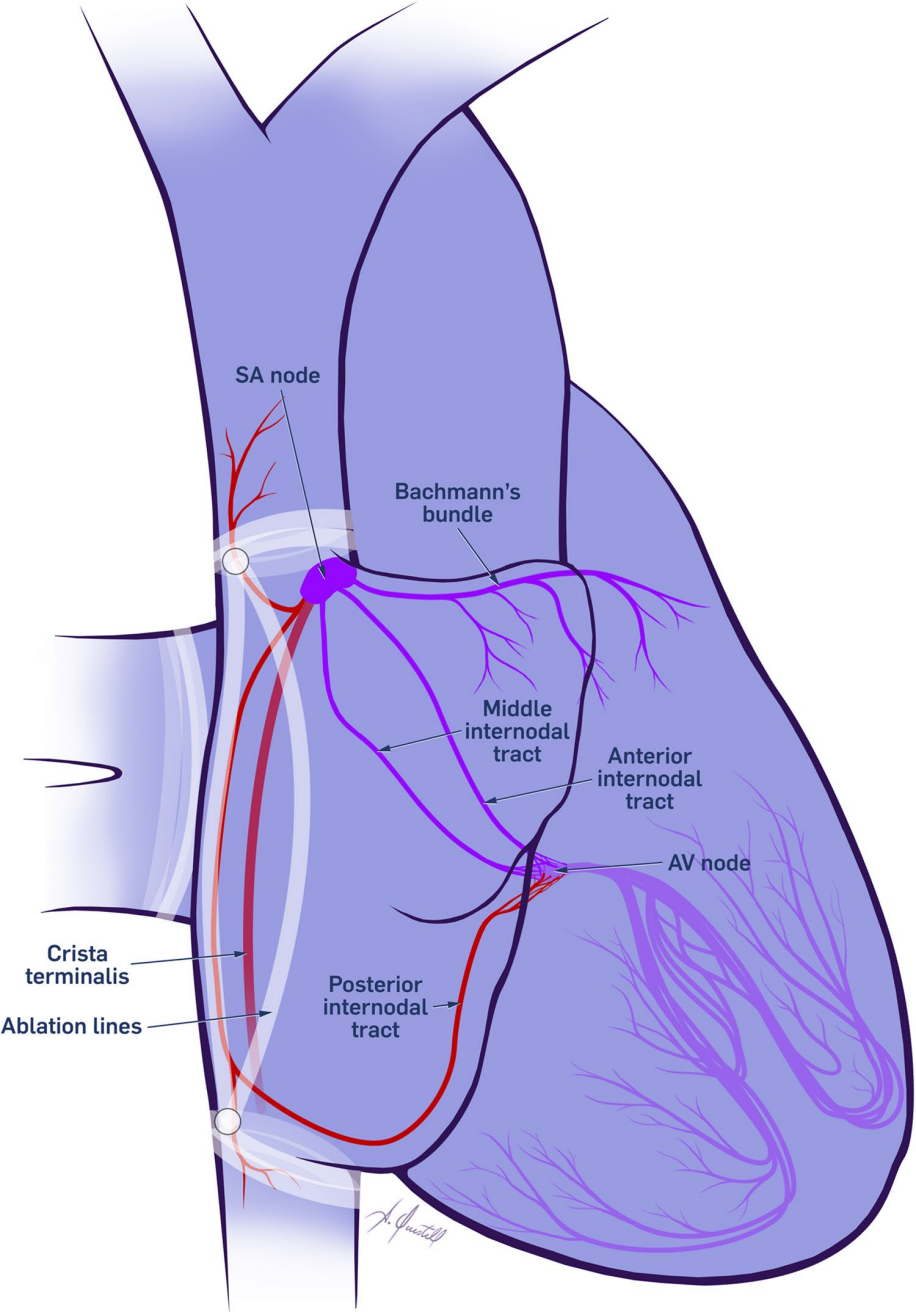
Schema of the right atrium (RA) in red the conduction system targeting during ablation, in blue the conduction system preserved during ablation, with a white line the schematic orientation of ablation line. SA node, sinoatrial node; AV node, atrioventricular node

Interestingly the P-wave morphology during tachycardia recorded by 12-lead ECG was nearly identical to that in sinus rhythm. At the beginning of the procedure, all patients with IST presented sinus tachycardia; no changes were observed during the induction of general anesthesia. In patients diagnosed with POTS, isoprenaline was administered in order to increase the sinus rhythm to 75% of maximal HR following the Sheffield formula (220-age/100) *75 bpm [12]. We performed simultaneous recordings and measurements with multi-electrode catheters from the SN in comparison with superior vena cava, crista terminalis, and inferior vena cava as shown in Fig. 3. Remarkably, the electrical activation recorded in all positions resulted on time with the early activation in the SN. Differential pacing from all regions demonstrated a post pace internal (PPI) of less than 5 ms in all sites investigated. We assumed that all regions were activated simultaneously to the SN. In our approach, we decided to disconnect the SN from the fast conduction system of the crista terminalis and the sleeves deep in the SVC and IVC as described in Fig. 3.

### Peri- and post-procedural complications

No complications were reported during the ablation procedures. During follow-up, pericarditis was the most common complication which occurred in 121 patients (47%) and was treated within the first 3 months. In 24 patients (9%), the symptoms continued up to 6 months, and standard treatment with methylprednisone, aspirin, and colchicine was administrated. Six patients (2%) had pleural effusions that resolved with medical treatment. Three patients (3%) had a severe pleural effusion that required surgical drainage. No blood transfusions were required. Pneumothorax was observed in 5 patients (1.9%), only 3 (1.1%) required surgical drainage. Five patients (1.9%) required a dual-chamber pacemaker due to sinus arrest > 5 s. Moreover, we noticed during pacemaker interrogation that in three (1.1%) of the five (60%) patients, atrial stimulation was utilized less than 1% of the time. A total of 13 patients (5%) required an additional catheter ablation procedure due to the presence of a right atrial tachycardia, in all patients normal sinus rhythm was restored (Table 3).

**Table 3:** Complication, follow-up. *n* = 255

Pericarditis, *n* (%)	121 (47)
Pericarditis up to 6 months, *n* (%)	24 (9)
Pleura effusion total, *n* (%)	6 (2.3)
Pleura effusion with surgical drainage, *n* (%)	4 (1.5)
Pneumothorax total, *n* (%)	5 (1.9)
Pneumothorax with surgical drainage, *n* (%)	3 (1.1)
Dual chamber pacemaker, *n* (%)	5 (1.9)
Ablation of atypical right atrial flutter/tachycardia, *n* (%)	13 (5)
Follow-up	
Follow-up (years)	4.07 ± 1.8
Mean hospital stay (days)	4.04 ± 0.37
Mean intensive care unit stay (days)	1.06 ± 0.09
Patient follow-up with loop implantable recording system, *n* (%)	224 (87)
Patient follow-up with serial Holter ECG, *n* (%)	31 (12)

### Follow-up

The mean follow-up after ablation was 4.07 ± 1.8 years. The 31 (12%) patients without implantable loop recorder monitoring were followed with serial Holter 24-h ECG monitoring every 3 months. After a blanking period of 6 months, 204 patients (80%) showed a significant reduction in HR compared to the pre-ablation period. In a subgroup of 198 patients (77%), the NN parameter increased from a mean 542.30 ± 28.60 to 1022.60 ± 169.01 ms. Additionally, the standard deviation of all normal RR intervals before ablation was 97.4 ± 14.07 ms and improved after ablation to 142.49 ± 40.65 ms. After the blanking period, all patients presented with a normal chronotropic response to exercise. Of note, none of the 51 patients (20%) with POTS experienced further syncopal episodes following ablation. All patients discontinued medication during the follow-up. We analyzed pre- and post-ablation electrocardiogram registrations in a subgroup of 198 patients (77%). (Table 4) The *P*-wave duration was shortened, and the first component of the *P*-wave (19.8 ± 45 ms) was missing post ablation (*P*-wave duration before 115.7 ± 58 ms and *P*-wave duration post ablation 96.8 ± 73 ms).

**Table 4:** Time-domain heart rate variability *n* = 198

	**Definition**	**Pre-ablation (*n* = 198)**	**Post-ablation (*n* = 198)**
Mean NN (ms)	Mean of all normal RR intervals (normal to normal coupling interval)	542.30 ± 28.60	1022.60 ± 169.01
SDNN (ms)	Standard deviation of all normal RR intervals (SDRR or CLV)	97.4 ± 14.07	142.49 ± 40.65
SDANN (ms)	Standard deviation of mean RR interval for all 5-min segments of 24-h ECG recordings	70.60 ± 21.69	128.21 ± 32.09
SD (ms)	Mean of standard deviations of all normal RR intervals for all 5-min segments of a 24-h ECG recording	23.73 ± 4.01	63.55 ± 13.71
rMSSD (ms)	Root mean square successive differences between adjacent normal RR intervals over the entire 24-h ECG recordings	16.79 ± 9.51	36.39 ± 10.01
pNN50 (%)	Percent of difference between adjacent normal RR intervals that are greater than 50 ms computed over the entire 24-h ECG recordings	5 ± 4.2	18 ± 8.2

## Discussion

Our multicenter findings demonstrated the feasibility and safety of a novel hybrid endocardial/epicardial ablation approach in IST/POTS patients for whom previous multiple therapeutic medical attempts were not successful. Due to the failure of medication in patients affected by IST/POTS, several invasive strategies relying either on endocardial or epicardial ablation approaches to modify the SN have been developed. However, most were hampered by partial success and high rates of complications. Since the SN is not a focal structure [13] in most cases, larger areas of the high right atrium need to be ablated in order to achieve appropriate HR reduction. Generally, the more superior parts of the SN, which are innervated by sympathetic stimulation, provide higher HRs. Whereas vagal stimulation has the tendency to activate more inferior portions of the SN resulting in slowing of the HR. The right PN runs laterally or posterior laterally to the superior vena cava and follows the lateral right atrium to the diaphragm (Fig. 1). Therefore, there are multiple potential sites of PN damage during endocardial ablation. To avoid this complication, high output pacing is typically used before radiofrequency application. If PN capture is present, energy delivery is avoided at these sites. The area of PN capture can be quite substantial, hence, during conventional catheter RF procedures, potential PN paralysis significantly limit success rates [14]. In addition, the regional anatomy of the SN and superior vena cava–right atrial (SVC–RA) junction is complex, with endocardial ridges that include the crista terminalis, the pectinate muscles, and the arcuate ridge, making endocardial ablation less successful. To overcome these limitations, a combined endo/epicardial approach has been developed, using percutaneous subxiphoid access to the pericardium and a balloon catheter for PN protection. Jacobson et al. presented a series of 5 patients who underwent combined endo/epicardial procedure after failed endocardial procedure [15]. Four patients were successfully treated and 1 had a recurrence which was treated by cryoablation via mini-thoracotomy. Three patients developed pericarditis after the ablation procedure. Another experience was reported by Shandling et al. on thoracoscopic ablation of SN, demonstrated the feasibility of utilizing microwave energy without electrophysiological mapping [16]. Other nonmedical possibilities for the treatment of IST include outdated surgical (on-pump) RA resection and SN isolation [17], AV node ablation and pacemaker implantation [18], and minimally invasive surgical approaches [19].

**Fig. 4:**
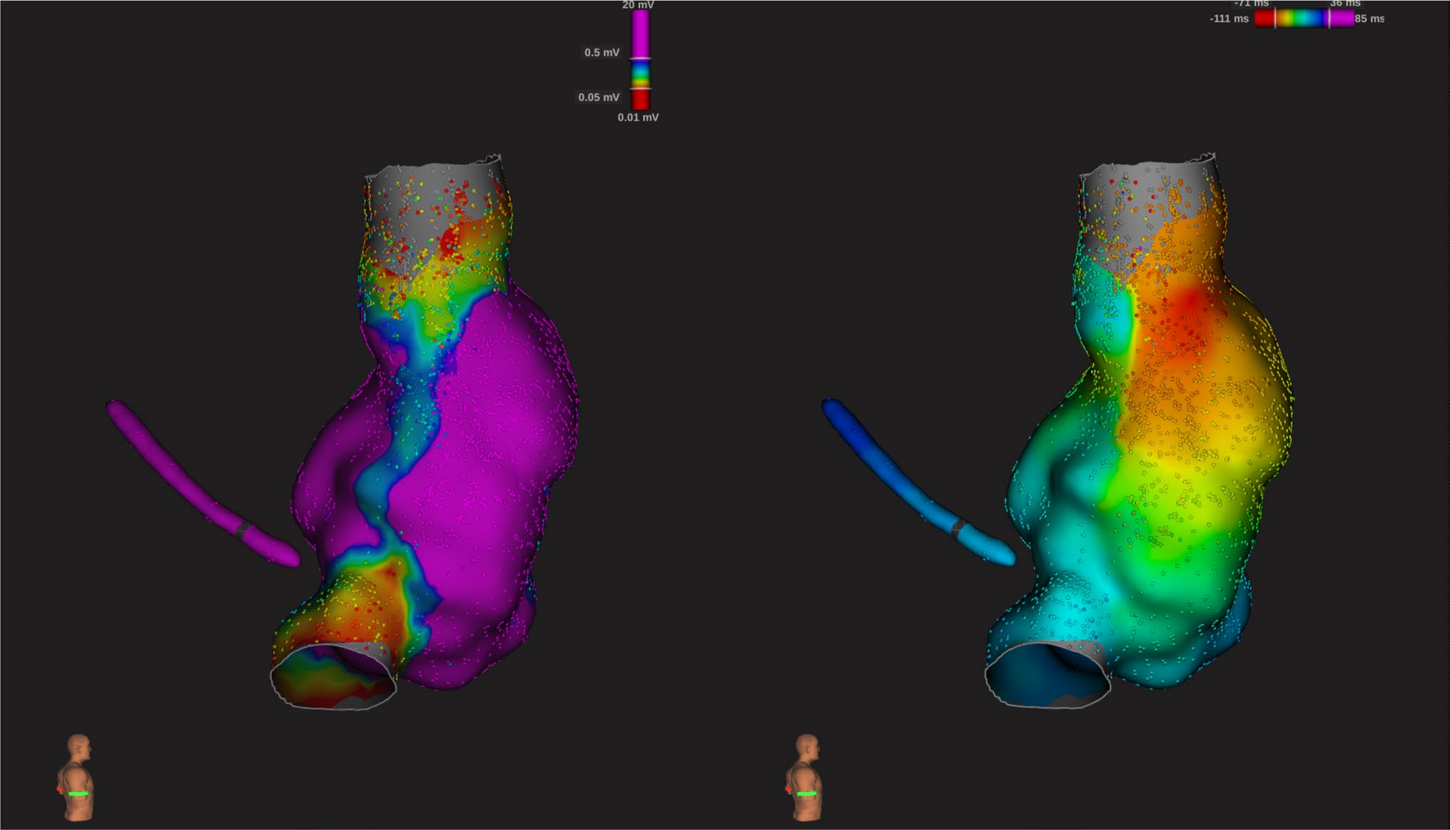
Example of post-ablation electroanatomical mapping. We can appreciate the isolation of the superior and inferior cava veins and the lateral line

**Fig. 5:**
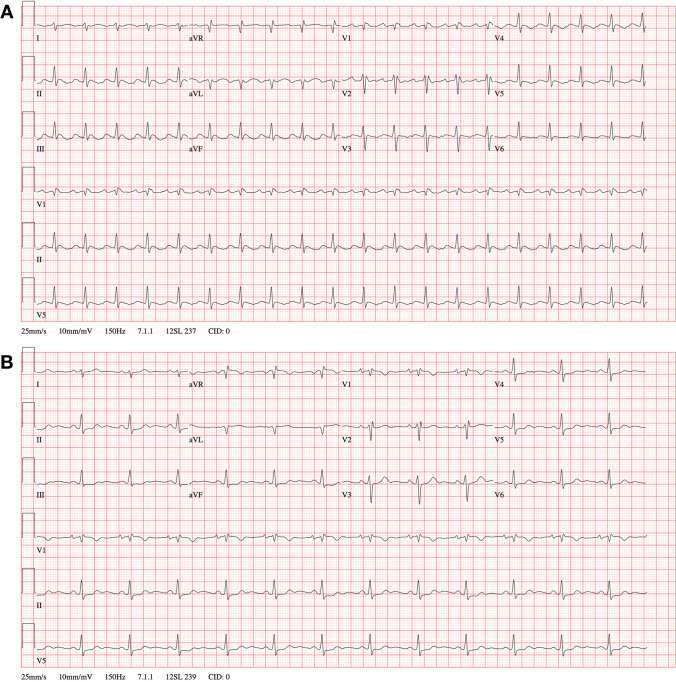
Example of electrocardiogram pre (**A** the patient was under general anesthesia) and post (**B** post procedure without anesthesia) procedure

**Fig. 6:**
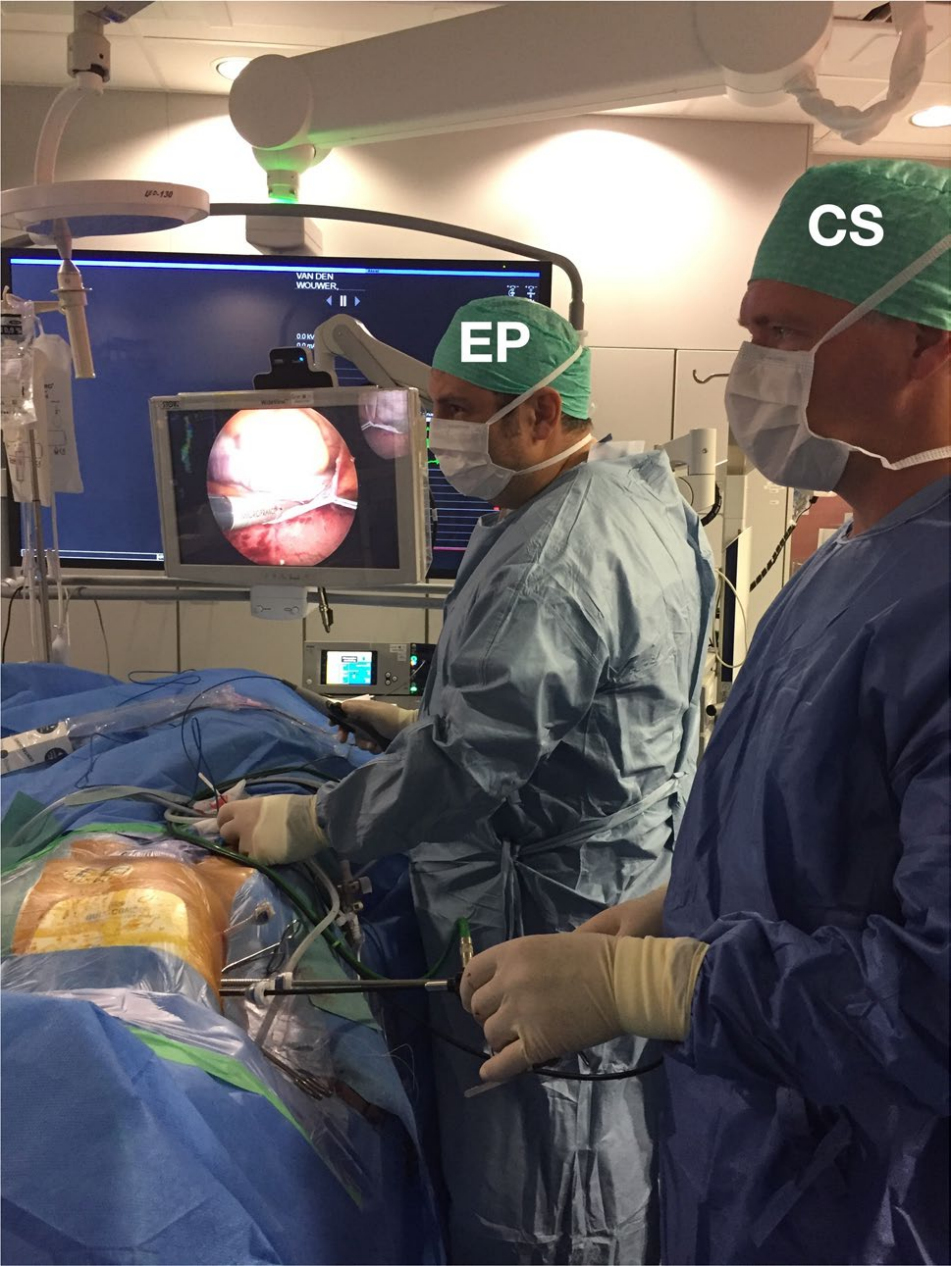
Hybrid simultaneous setting. EP, electrophysiologist; CS, cardiac surgeons

**Fig. 7:**
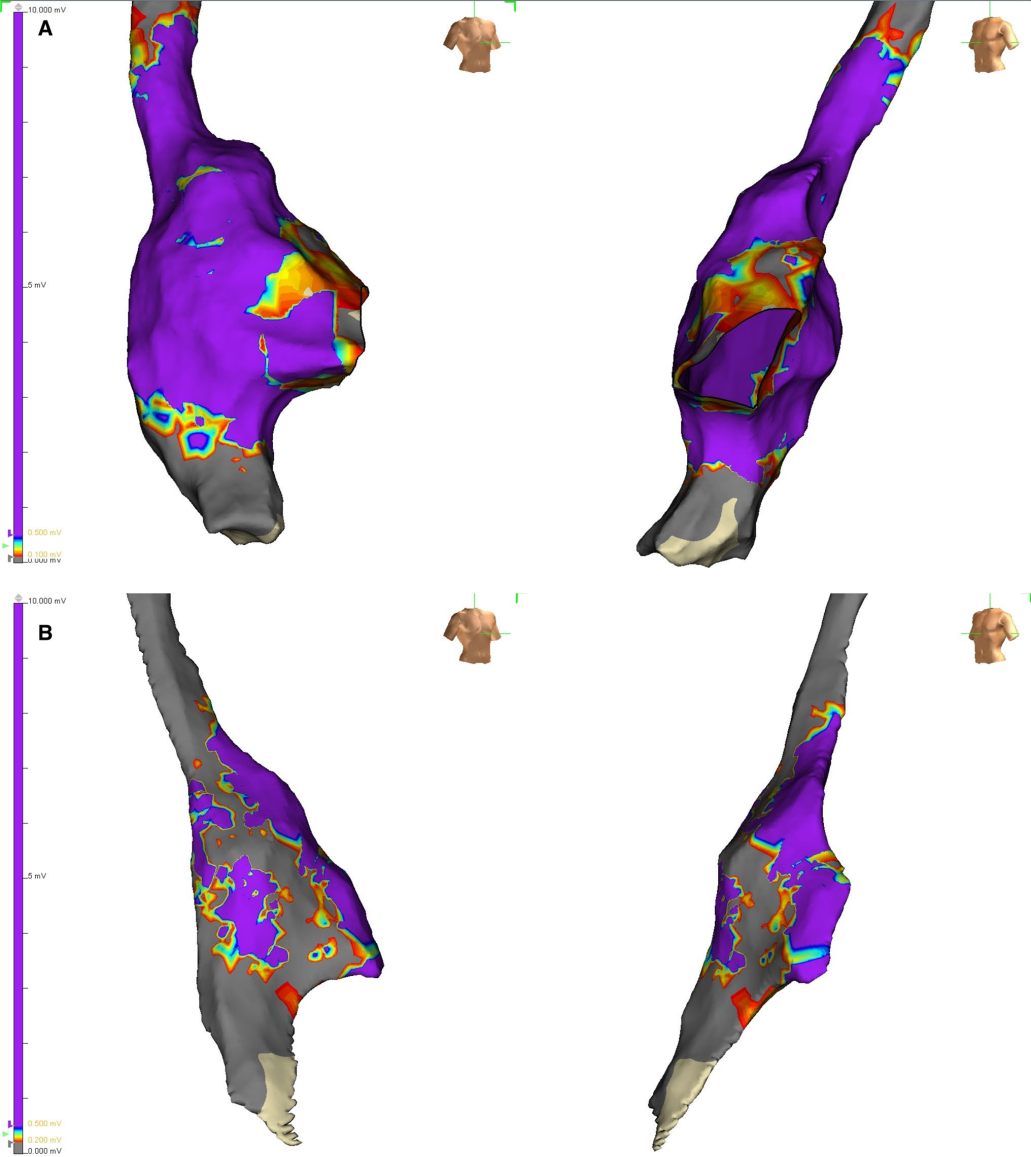
Example of pre (panel **A**) and post (panel **B**) mapping

The hybrid approach described has a number of distinct advantages over currently used ablation strategies; (i) minimal invasiveness, other than a femoral venous puncture, and three (5-mm) ports are placed on the right chest; (ii) direct visualization of the structures of interest and better direct energy delivered; (iii) minimal risk of PN damage; (iv) simultaneous endocardial activation mapping allowing SN identification and precise epicardial ablation (Fig. 4). Also, the bipolar RF clamp can be closed on the area of interest without ablating, which will simulate an ablation and will ensure that a safe position near the sinus node is attained. To the best of our knowledge, this is the largest series of patients treated for IST/POTS with ablation to date. In our multicenter experience, at the end of the procedure, a majority of patients presented a significant decrease in HR, RR intervals, and HRV. This was maintained during the entire follow-up (mean follow-up of 4 years). Considering the strongly debilitating symptoms prior to ablation, these excellent results justify this minimally invasive approach. Importantly, an epicardial procedure will provide direct visualization of the PN, therefore reducing the potential for injury (Fig. 5). Finally, endocardial mapping allows for electrophysiologic guidance to an otherwise “blinded” anatomic approach. Although minimally invasive, our approach may have a higher potential rate of complications compared to conventional endocardial ablation. There is the risk of general anesthesia, bleeding, pneumothorax, and damage to cardiac and extra-cardiac structures during a thoracoscopic procedure that could result in sternotomy and/or conversion to open-heart surgery. These complications have already been described in a larger series of patients undergoing hybrid ablation for AF [10]. Therefore, given the young age of this patient population and the invasive nature of ablations, we do not recommend that they be part of routine care as first-line therapy. Of note, in our procedure, there might be a lower incidence of complications when compared to thoracoscopic ablation of other arrhythmias, since only a right-sided approach is used. In addition, the procedure time is shorter, and patients are not anticoagulated. In fact, our patients did not experience any life-threatening complications. The most common adverse event was pericarditis. Although predictable, it could be difficult to treat in some cases. In our cohort, all symptoms of pericarditis were resolved with medical treatment. The patients affected did not bear any sequelae at final follow-up related to this complication. The procedure was performed by experienced operators with vast experience of thoracoscopic epicardial AF ablation (Fig. 6). Although the complication rate may have been higher in less experienced hands, we believe that hybrid ablation for IST/POTS, using surgical thoracoscopic video-assisted epicardial ablation combined with endocardial 3D mapping, may be considered an effective and safe option for symptomatic patients who have failed medical therapy (Fig. 7).

Finally, we believe that this approach might lead to a better understanding of the complex structure of the SN, both from an anatomical point of view and from an electrophysiological standpoint. Currently, there is no specific training that is required before a surgeon performs surgical ablation. It may be necessary to establish credentialing criteria for surgeons wanting to perform surgical ablation with novel technologies, including both proctoring and mentoring protocols in the operating room. Training and mentoring are essential for this technique to be implemented with the best possible outcomes for patients. Following the expert consensus, we recommend that surgeons who are new to surgical ablation be proctored by an experienced surgeon for 3 to 5 cases before performing surgical ablation alone. In terms of maintenance of proficiency level, surgeons with sufficient training should aim to routinely perform surgical ablation cases [9].

## Limitations

Some limitations can be found in our study. The present work was a retrospective study. Most of the electrocardiographic markers analyzed in the study are dynamic and the real prevalence of these parameters is difficult to evaluate. Larger, prospective, and multicenter studies are needed to confirm our findings. We did not perform any official “quality of life” testing, which should be considered moving forward.

## Conclusions

A novel sinus node sparing hybrid ablation for treatment of IST/POTS, using surgical thoracoscopic video-assisted epicardial ablation combined with endocardial 3D mapping, appears to be a successful approach. This promising treatment for patients with symptomatic IST and POTS offers a complete restoration of the normal HR and HRV, with a total reduction of all symptoms. Our experience was free from major complications, had a very small number of pacemaker implantation, and had no PN injury. Interestingly in our cohort, this hybrid treatment has offered an option to patients who have struggled to find any other relief from this debilitating disease, its symptoms and its psychosocial consequences.

## Abbreviations

POTS: Postural orthostatic tachycardia syndrome
IST: Inappropriate sinus tachycardia
bpm: Beats per minute
SN: Sinus node
HR: Heart rate
HRV: Heart rate variability
PN: Phrenic nerve
